# A novel natural scaffold layer improving efficiency, stability and reproducibility of Perovskite solar cells

**DOI:** 10.1038/s41598-023-31366-5

**Published:** 2023-03-15

**Authors:** Esma Yenel, Mahmut Kus

**Affiliations:** 1 Department of Electricity and Energy, Technical Science Vocational School, Konya Technical University, Konya, Turkey; 2 Department of Chemical Engineering, Faculty of Natural Science and Engineering, Konya Technical University, Konya, Turkey

**Keywords:** Chemistry, Energy science and technology, Materials science, Optics and photonics, Physics

## Abstract

In this study, our hypothesis was to demonstrate the usability of a natural clay structure as scaffold layer in perovskite solar cells (PSCs). Sepiolite, which is a natural and environmentally friendly clay structure, has a very high active surface area and can easily be dispersed in solvents. In addition we predicted that crystallization could easily occur on their surfaces due to their surface chemistry. In the study, we firstly used a natural clay as scaffold layer in PSCs. It is observed that, efficiency, reproducibility and stability of PSCs have been significantly improved. Improvements in efficiency have been observed to be between 30 and 50% depending on the type of perovskite solvent used. In addition, the surface chemistry of the sepiolite resulted in better crystallization as well as stability. Due to its high-water adsorption capability, sepiolite makes the perovskite crystal more stable by trapping the residual water molecules as well as penetrated water molecules from environment. Consequently, we demonstrated that, a natural, low-cost and environmentally friendly clay may be an alternative material which may contribute to the commercialization of PSCs.

## One-sentence summary

May a natural clay sepiolite be a solution for stability and reproducibility of perovskite solar cells?

Perovskite solar cells (PSCs) became one of the most important solar cell technologies currently being studied by many researchers due to its high efficiency, low-cost and facile fabrication. In general, the structure of perovskite solar cell is introduced to be a sandwich structure of perovskite layer as light absorber between a hole and an electron transport layers. Two different architectures, as planar and mesoscopic structures are known in literature. In planar structure each layer, including perovskite layer, are formed as dense films sequentially. In mesoscopic structure, perovskite layer is adsorbed on a mesoscopic structure called as scaffold layer^1,2^.Optimization of all layers, such as formation of pin hole free perovskite layer, large crystal grains, improving interface matching between p-i-n layers, is the main focus for planar PSCs. In case mesoscopic PSCs, independent from the chemical structure (TiO_2_, SiO_2_, Al_2_O_3_, NiO), scaffold layer limits the size of perovskite crystal. Nevertheless, mesoscopic structure allows thicker perovskite layer than planar structure for better light harvesting and efficiency^3^. In early stage, mesoscopic structures were n type materials such as TiO_2_ and ZnO^4,5^. In 2012, Lee et al. reported an insulator scaffold layer (Al_2_O_3_) resulting in 10,9% efficiency in PSCs^6^. In this work, they presented that perovskite layer is able to electron transfer beside light absorption. Thus, a new description as active and passive scaffold layer is introduced in literature. If scaffold layer plays a role in electron transfer, it is described as active, in case no role in electron transfer, it is passive scaffold layer. TiO_2_, ZnO, NiO and SnO_2_ are already reported materials as active scaffold layers, while SiO_2_, Al_2_O_3,_ ZrO_2_ are passive scaffold layers in literature^1,7,8^. One of the most important problems for mesoporous scaffold layer can be described to be the time requirement for deposition of perovskite solution in meso structure. Hwang et al. investigated influence of different sized SiO_2_ nanoparticles as scaffold layer on performance of PSCs^9^. They observed 50 nm particle size gives the best performance due to the better penetration of solution and thus better crystallization of perovskite layer. On the other hand some researchers suggested nanorods instead of nanoparticles as scaffold which allows better pore filling and results in better performance^10,11^. Lee et all reported a comparative study on influence of nanorods and nanoparticles of SiO_2_ on performance of PSCs and observed that nanorods gives better crystallization and pore filling of perovskite the results in better performance^12^. Some other researchers focused on modification or doping of mesoporous TiO_2_ layer. For instance, Dao et al. investigated HCl treatment of mesoporous TiO_2_ layer to enlarge pore size and passivate hydroxy groups to improve the performance of PSCs^13^. Omrani et all used plasmonic nanoparticles for energy level alignment to enhance the performance of mesoporous PSCs^14^ They observed significant improvement in efficiency by using Ag-SiO_2_ and SiO_2_-Ag-SiO_2_ plasmonic nanoparticles. Not only penetration problem but also photocatalytic activity of some scaffolds can be described as another problem must be solved for such systems^15^. Although PSCs are cost effective and efficient, there are still problems must be solved such as reproducibility and long-term stability for large area fabrication and commercialization of this technology. So, introducing novel approaches such as a novel scaffold layer which provides some solutions for the problems mentioned above. Using natural clays in PSCs as scaffold layer sounds good due to their many advantages such as natural and nontoxic features. We reached three papers related to natural clays and perovskite solar cells. The first paper reported by by Li et al.^16^. They reported that montmorillonite as a buffer layer between perovskite and hole transport layer and observed significant improvement in stability. The second paper is reported by Huang et al.^17^. They used montmorillonite as additive in perovskite layer around 0.01%wt and observed significant stability enhancement in PSCs. The third one is reported by Mokhtar et al.^18^. They added spherical hydroxyapatite nanoparticles in TiO_2_ scaffold layer to capture reduced lead release from damaged PSCs. Beside the adsorption of released lead from perovskite layer by spherical hydroxyapatite, improvement in solar cell efficiency has been clearly observed.

In this work, for the first time, we introduce a novel scaffold layer improving stability, reproducibility and efficiency of PSCs. Moreover, this novel scaffold layer, sepiolite, is fully natural and can be used directly without no further chemical process. Sepiolite is a natural mesoporous clay with a unit cell formula Si_12_O_30_Mg_8_(OH,F)_4_(H_2_O)_4_▪8H_2_O having extremely large surface area due to its’ mesoporous fiber like structure^19^. Sepiolite fibers includes microtubular structures having dimensions about 1.06 × 0.37 leading high pore volume and extremely high adsorption capacity^20,21^. Beside large surface area, fiber like structure and consistence of hydroxyl groups make them attractive materials for many applications^22,23^. Fiber like morphology leading large surface area and mechanical stability, earth abundant and green chemical structure, functional groups on its’ surface and thermal stability of sepiolite motivated us to use it as scaffold layer in PSCs. Except for our patent applications we have not reached any report related with natural clays as scaffold in PSCs^24^.

Interestingly, sepiolite as scaffold layer leads to improve efficiency between 30 and 50% depending on perovskite precursor solvent (Acetonitrile, gamma butyrolactone or dimethyl sulfoxide) in comparison with reference PSC. Moreover, sepiolite as scaffold layer improves reproducibility due to natural crystal structure of clay which leads to formation of large perovskite crystal grains and long-term stability due to its’ high adsorption capability of moisture leading to prevent the diffusion of water molecule through to perovskite crystal. These results are very important for large area fabrication of stable and reproducible PSCs. This paper also provides the researchers new ideas for novel natural scaffold layers.

## Results and discussion

**Figure 1:**
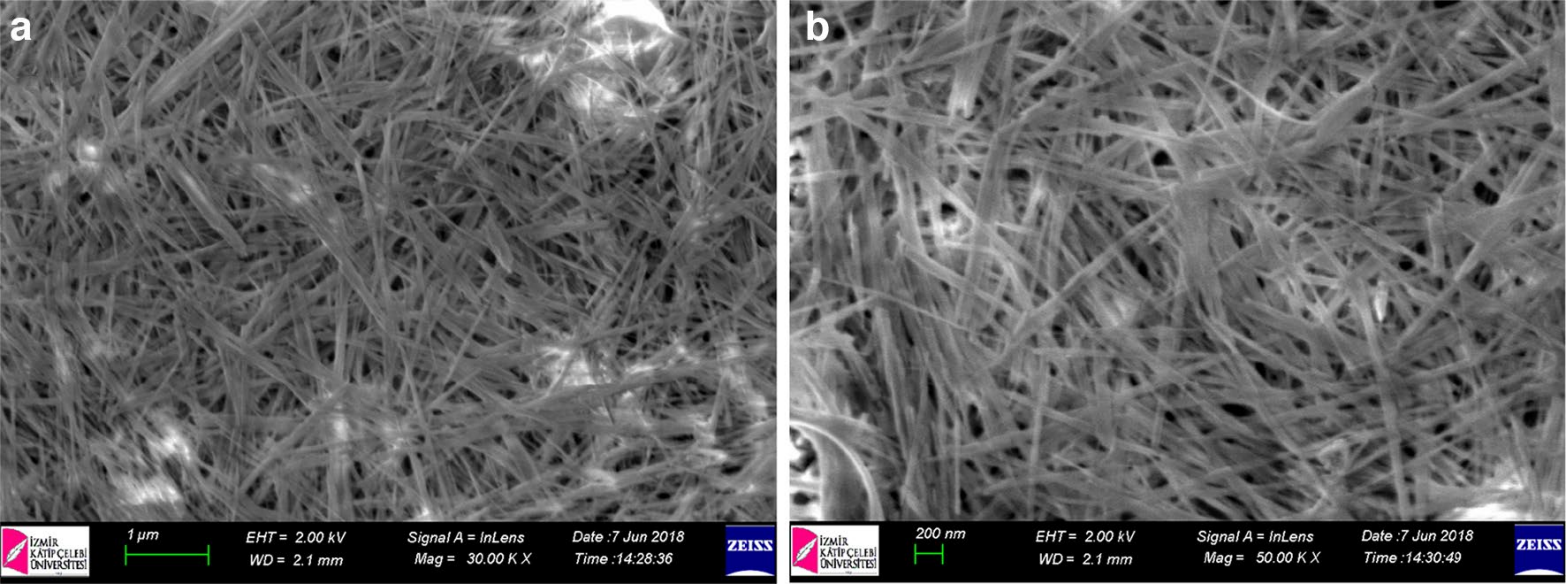
SEM image of aerosol coated sepiolite films on FTO glasses obtained from 1 mg/ml dispersion in water. (**a**) 30KX and (**b**) 50KX magnification.

Sepiolite is a natural fiber-like mesoporous materials having an extremely large active surface area. In this work we used sepiolite as scaffold layer in perovskite solar cells. At the beginning, we investigated the best solvent system and film preparation of sepiolite films. DMF, DMSO, GBL, water, acetonitrile, isopropanol and ethanol are used as solvent for sepiolite (see supporting info in Fig. S1). Due to strong hydrophilic feature of sepiolite gives the best dispersion with water as expected. Water completely dissolves sepiolite and gives a gel-like dispersion which is easily useable for spin cast or aerosol coating. The viscosity of gel-like dispersion can be tuned for thickness optimization of sepiolite film. After thickness optimization we observed that 0.1 mg/ml water dispersion of sepiolite gives the best film (homogenous) and thickness (~ 500 nm see Fig. S2). Figure 1a-b shows the SEM image of sepiolite films coated on FTO glasses. It can be obviously observed from the figure, sepiolite fibers are homogenously distributed on FTO glasses. Total thickness of sepiolite layer is measured to be around 500 nm. After perovskite coating total thickness is measured to be around 600–650 nm (provided by profilometer) which may be considered as an acceptable value for perovskite layer in PSCs^25,26^.

We must indicate here that we used planar perovskite solar cells as reference due to low efficiency of m-TiO_2_ PSCs in comparison with sepiolite included perovskite cells (S-PSCs). We observed maximum 7.92% efficiency for m-TiO_2_ PSC while it is around 16% for S-PSCs for the same experimental conditions (see Table 1 and Fig. S3). However, the efficiencies for planar PSCs as reference are more comparable than that of m-TiO_2_ PSC.

**Table 1:** IV characteristics of Sep-PSCs and their references based on precursor solvent.

	I_sc_ (mA/cm^2^)	V_oc_ (mV)	W_max_ (Mw/cm^2^)	FF	Efficiency (%)
R-PSC GBL	18,8	950	9,1	0,51	11,4
S-PSC GBL	21,3	950	12,9	0,64	16,1
R-PSC ACN	16,5	900	8,0	0,53	9,94
S-PSC ACN	21,2	900	10,2	0,54	12,8
R-PSC DMF:DMSO	15,3	950	7,5	0,51	9,4
S-PSC DMF:DMSO	21,6	950	11,9	0,57	14,8
m-TiO_2_ PSC	12,1	850	4,8	0,46	7,9

**Figure 2:**
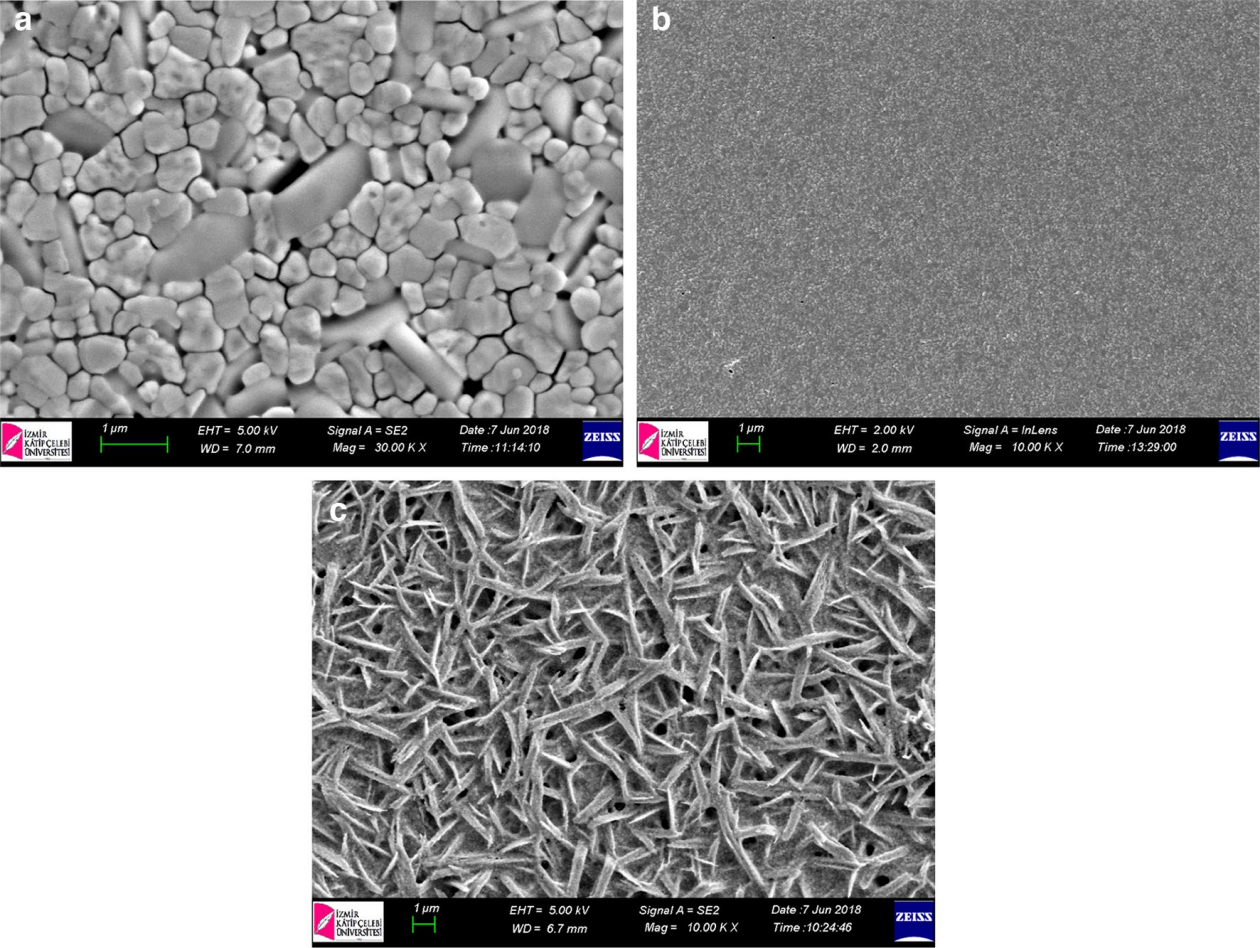
(**a**–**c**) SEM images of perovskite layer on sepiolite scaffold prepared from (**a**) ACN (**b**) GBL and (**c**) DMF:DMSO solutions.

After optimization of sepiolite mesoporous layer preparation, we focused the type of solvent for perovskite precursors such as ACN, GBL and DMF:DMSO. As well known, ACN is a solvent which leads rapid crystallization of perovskite and thus results in reproducible devices^27^. Fast evaporation of solvent is a disadvantage for mesoporous scaffold due to limited time for penetration of perovskite solution in meso structure. However, this is not valid when sepiolite used as scaffold. We obtained around 12% efficiency which in agreement with planar structures in literature^27^. This is due to large pore size of sepiolite fibers which allows fast penetration of precursor solution into sepiolite film. Figure 2a–c shows SEM images of perovskite layer on sepiolite scaffold prepared from a) ACN b) GBL and c) DMF:DMSO solutions.

**Figure 3:**
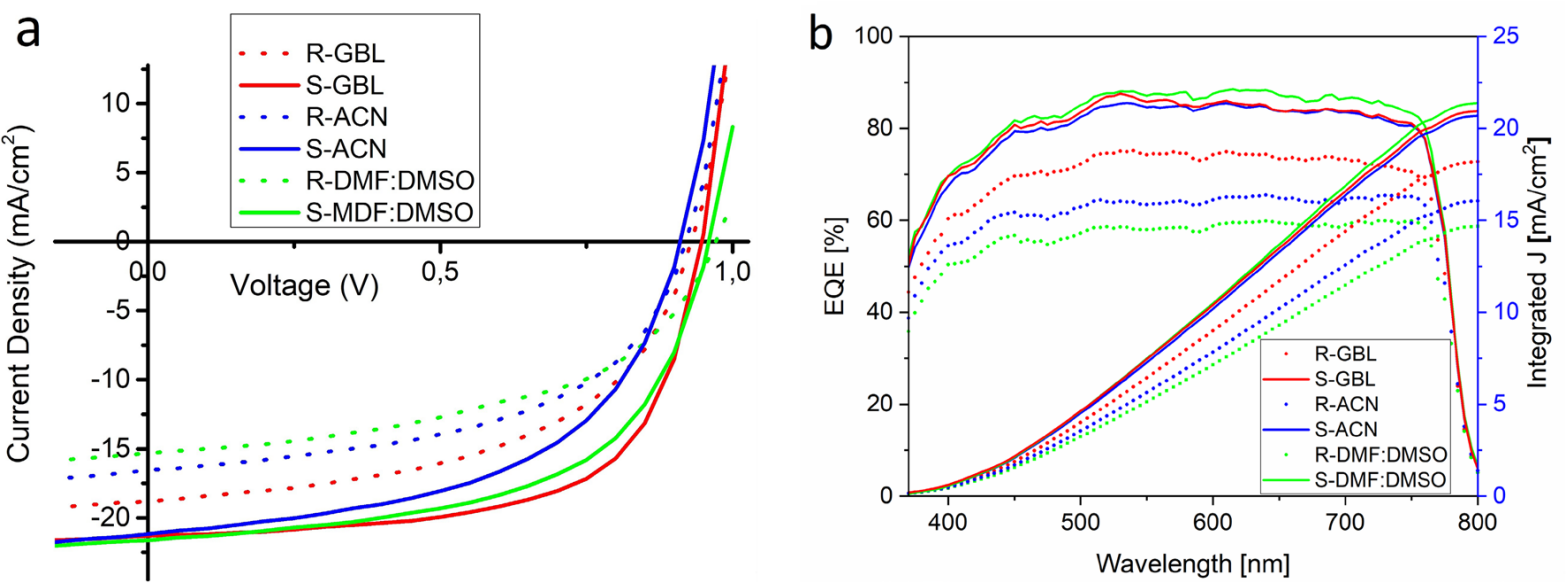
(**a**) Comparison of IV results for solvent types and their planar reference PSCs and (**b**) IPCE results for each PSCs. (R and S refer to reference PSCs and Sepiolite included PSCs respectively. GBL, ACN and DMF:DMSO are solvents used for perovskite layer preparation).

SEM images show that ACN (Fig. 2a) gives larger grains and thicker perovskite layer on sepiolite while the smoothest films are obtained with GBL (Fig. 2b). Here, DMF:DMSO gives an interesting structure that is something like surrounding of sepiolite fibers most probably due to high concentration of perovskite precursors leading very fast precipitation during spinning. Nevertheless, all solvent systems give more or less comparable improvements according to their reference PSCs, In comparison with reference device prepared from ACN solution, sepiolite scaffold improves efficiency around %30 (see Fig. 3a). On the other hand GBL and DMSO:DMF are other alternatives for perovskite precursors. Both systems require antisolvent washing for better efficiency. Similar with ACN, GBL and DMSO:DMF solvent system also work with sepiolite scaffold. The most attractive results were obtained with DMSO:DMF system which gives approximately 50% improvement in efficiency in comparison with reference device while this improvement is around %30 for GBL solvent. However, it must be indicated that GBL solution give more reproducible results in comparison with the other perovskite precursor solutions. Figure 3a shows comparison of IV measurements for all solvent system with their planar reference PSCs.

Table 1 summarize IV characteristics of references and Sep-PSCs. In Table 1, R means reference and S means sepiolite scaffold included, and GBL, ACN, DMF:DMSO indicates perovskite precursor solvents. Table also includes characteristics of meso TiO_2_ as another reference.

When sepiolite used as scaffold, PSCs show no change for V_oc_ values in comparison with their references. FF values show improvement for GBL and DMF:DMSO precursors but not for ACN precursor. However, I_sc_ values show improvement for all precursors (see Table 1). To better understand these observations, we have to focus on penetration of precursors into scaffold layer and crystal formations. As well known, penetration of perovskite precursors in meso structure is critical issue for mesoporous PSCs^9^. Better penetration results in better crystallization and efficiency. In our work, in case sepiolite used as scaffold, penetration time is not necessary while it is a must for m-TiO_2_. Fiber like structure of sepiolite having large pores lets perovskite solution to penetrate inside to whole meso structure and results in significant improvement in efficiency. Moreover, this structure also allows better drying of active layer during post treatment resulting in better crystal structure. Of course, not only penetration capability but also better drying cannot explain those significant improvements in efficiency. As very well-known crystal quality, grains and grain boundaries play critical role in the performance of PSCs. Sepiolite also has a crystal structure including mostly Si and Mg oxides leading OH rich surface feature on its own grains. Such surfaces are very useful for crystal formation and growth. Xue et al. reported that amino and hydroxy groups on PEDOT:PSS surface facilitate the formation of perovskite crystals by coordinating uncoordinated Pb^2+^ and also decreasing surface defects^28^. Beside better crystal formation, hydroxy groups also improve the efficiency. Some of researchers reported that hydroxy terminating groups coordinate to uncoordinated Pb^2+^ leading to decrease in recombination^29,30^. The effect of hydroxy group on better crystallization and improving efficiency is also shown by Bai et al. for NiO/perovskite interface^31^. Consequently, sepiolite due to its crystal surface having so many hydroxy groups lead many nucleation centers for better crystallization of perovskite as well as improving efficiency by coordinating the uncoordinated Pb^2+^ and passivating the surface states. XRD results support our explanations about better crystallization of perovskite layer (see Fig. S4). Beside the efficiency, significant improvements on reproducibility and hysteresis are also support this suggestion which will be discussed in the next paragraph.

Figure 3b shows IPCE results for each PSCs fabricated from different solvents. All absorption peaks and plateau of IPCE results are similar but show differences in intensity depending on current density of each cell. The absorption of cells starts from nearly 400 nm at a level of 50% and reach up to 750 nm at a level of 80%. Although absorption of cell appears starting from 400 nm, in fact, maximum absorption intensity is observed at 450 nm which remains constant up to 750 nm indicating typical methylammoniumleadiodide (CH_3_NH_3_)PbI_3_ perovskite structure. It is clear from Fig. 3b, perovskite layer absorbs whole visible region and calculated current intensities are in agreement with current intensities observed on IV graphs.

Moreover, sepiolite scaffold leads to decrease in hysteresis (see Fig. S5). The hysteresis effect in PSCs is caused by many reasons such as traps in perovskite interfaces, ferroelectric polarization, ion migration/displacement or capacitive effects^32^. Lee et al. reported that perovskite provides charge transmission on its own crystal and transfers the charge directly to the anode material when an insulating material such as Al_2_O_3_ used as scaffold^33^. This explanation actually points out that the use of insulator material instead of semiconductor mesoporous TiO2 will eliminate the hysteresis effect. Yu et al. stated that the hysteresis effect decreases by using SiO_2_ (insulator) scaffold in comparison with m-TiO_2_ (semiconductor)^34^.

Absorption, transmittance and photoluminescence of perovskite layer on sepiolite, m-TiO_2_ and only perovskite (no scaffold) and also for only sepiolite are given in supporting information (see Fig. S6a-d). Absorption and transmittance shapes of perovskite layers show absorption band edge around 780 nm which is fully in agreement with previous reports^35^. On the other hand, remarkable increase in the intensity of photoluminescence of perovskite layer on sepiolite is observed in comparison with m-TiO_2_ and reference perovskite (no scaffold). The increase in photoluminescence intensity sourced from decrease of trap states on perovskite layer^36^. We attribute that sepiolite leads decreasing the trap states during the crystallization of perovskite layer. Jana et al. observed that perovskite layer on some clays show longer fluorescence lifetime and increase in intensity^37^.

**Figure 4:**
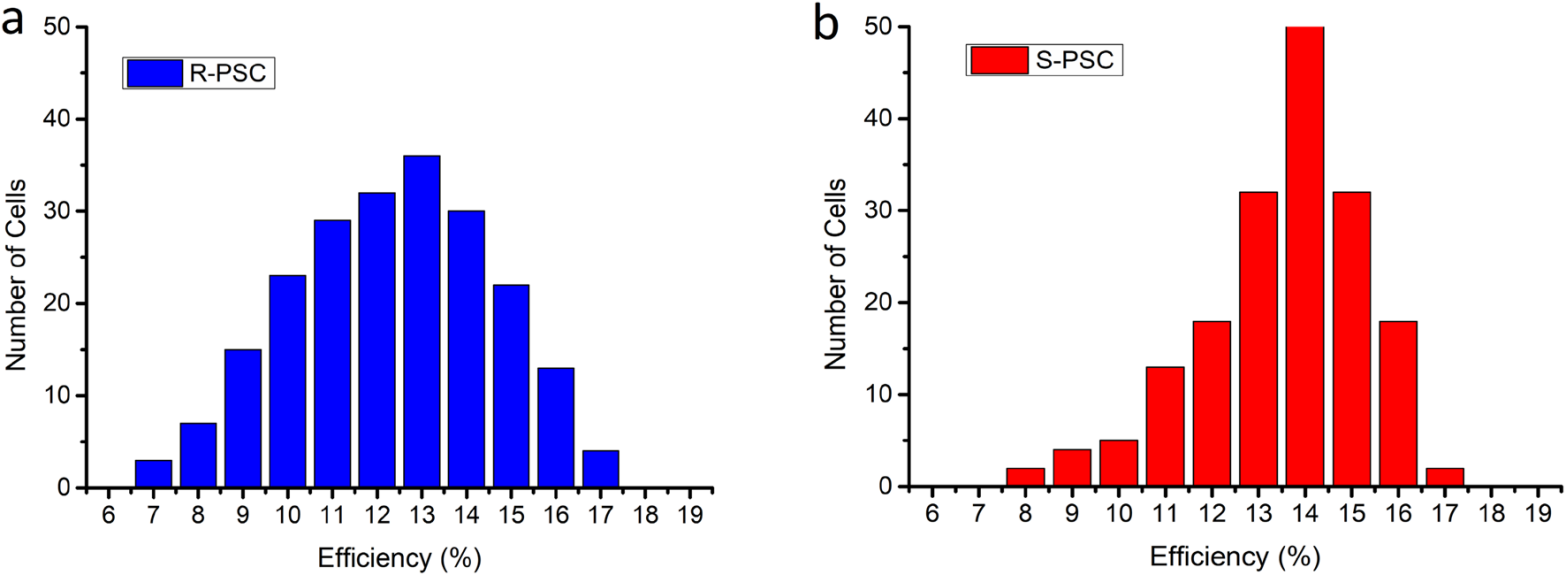
Average efficiency distribution of (**a**) reference (**b**) sepiolite included PSCs.

Figure 4a-b shows average efficiency distribution of reference and sepiolite included PSCs.

**Figure 5:**
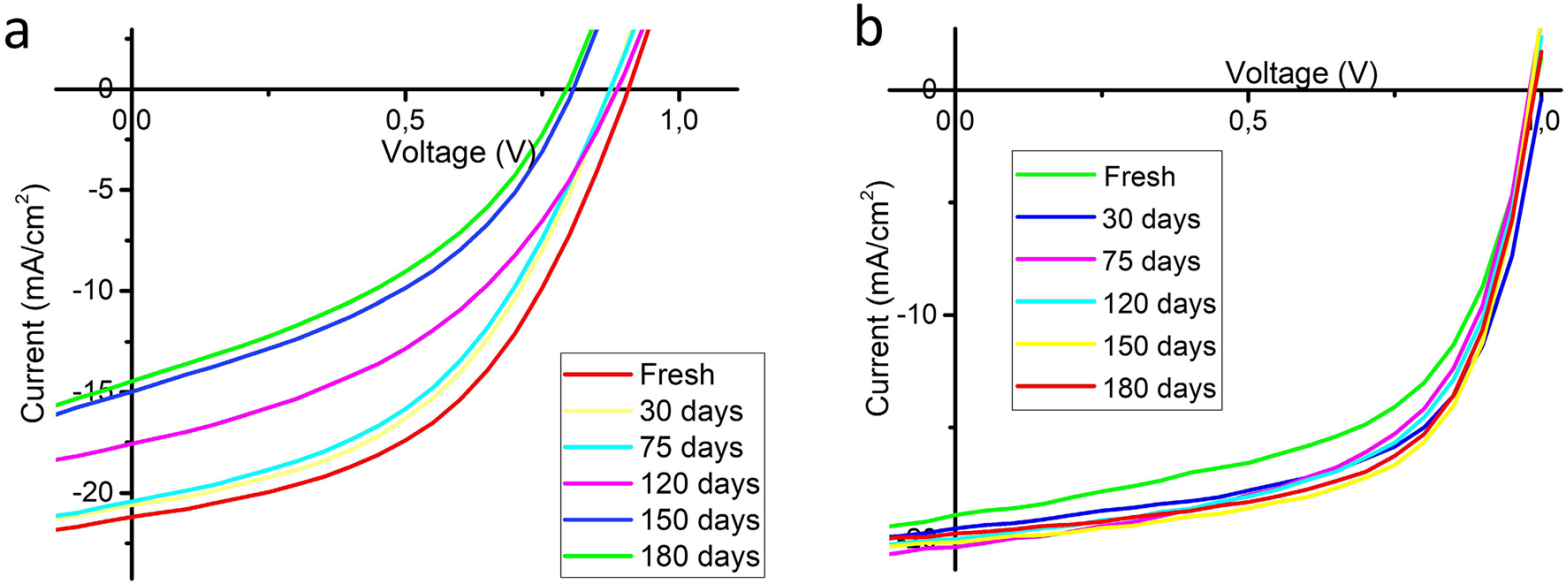
IV graph of (**a**) reference (**b**) sepiolite included PSCs for 6 months.

Reproducibility tests were carried out by fabricating more than 150 cells for both reference (R-PSC) and sepiolite included PSCs (S-PSC) under the same conditions. As it is clear from figure efficiency distribution becomes clearly narrower for sepiolite included PSCs than reference PSCs. This observation shows sepiolite not only improves efficiency but also increases reproducibility which seems to be a good advantage for large area fabrication of PSCs. But of course, beside the efficiency and reproducibility, stability is one of the most important issues for large area fabrication of PSCs. For stability test, we measured reference and sepiolite included PSCs at the same time intervals in glove box. Figure 5a-b shows IV graph of (a) reference (b) sepiolite included PSCs for 6 months.

It is clear from Fig. 5a-b, sepiolite significantly increases the stability of PSCs. The most interesting observation in Fig. 5b is the increasing of efficiency after freshly fabricated PSC. Efficiency increases from 17.6 to ~ 20% for freshly fabricated PSCs and 30 days aged PSCs. This observation may be explained as adsorption of water molecule residuals by time in perovskite crystals by sepiolite fibers due to the strong water adsorption capability of sepiolite. This causes an increase in efficiency in comparison with fresh and aged PSCs. Then the efficiency stays around 19–21% for 180 days measurements. As mentioned in introduction section Huang et al. used montmorillonite as additive in perovskite layer around 0.01%wt and observed significant stability enhancement in PSCs. They attributed that montmorillonite behaves as a protective shell that inhibits the moisture from penetrating into the perovskite during environmental aging^17^. On the other hand Li et al. reported that montmorillonite as a buffer layer between perovskite and hole transport layer prevents corrosion of perovskite structure^16^.

Consequently, a natural clay sepiolite as scaffold layer in PSCs leads efficient, reproducible and stable PSCs. Nontoxic chemical structure, low-cost availability in nature and facile processability of sepiolite may encourage researchers for large area fabrication of PSCs. All experimental data and some additional information (Fig. S7–S11) are available in supplementary documents.

## Supplementary Information

Supplementary Information.

## Data Availability

All data are available in the main text or the supplementary materials”.
