# Supplementary material for: A novel natural scaffold layer improving efficiency, stability and reproducibility of Perovskite solar cells
Source: Sci Rep. 2023 Mar 15;13:4319. doi: 10.1038/s41598-023-31366-5 (PMC10017716; doi:10.1038/s41598-023-31366-5)
Supplement: Supplementary file 1 — Supplementary Information. [file 41598_2023_31366_MOESM1_ESM.docx]

SUPPORTING INFORMATION

A Novel Natural Scaffold Layer Improving Efficiency, Stability and Reproducibility of Perovskite Solar Cells

Esma Yenel^1^*, Mahmut Kus^1^*

^1^Department of Electricity and Energy, Konya Technical University, Konya, Turkey

^2^Department of Chemical Engineering, Konya Technical University, Konya, Turkey

*Corresponding authors. Email: [esmayenel@gmail.com](mailto:esmayenel@gmail.com), [mahmutkus1@gmail.com](mailto:mahmutkus1@gmail.com)

Experimental section

Glass/FTO was purchased from KINTEC Corp. (with surface resistance 5-7 ohm). Titanium isopropoxide, acetylacetone, lithium bis(trifluoromethanesulfonyl)imide (Li-TFSI), 4-tert-Butylpyridine and solvents were purchased from Merck, phenyl-C61-butyric acid methyl ester (PCBM), Spiro OMETAD were purchased from LUMTEC, perovskite precursors, CH_3_NH_3_I (MAI), PbI_2_, were supplied from TCI, Sepiolite was provided by Sigma-Aldrich and TiO_2_ paste was purchased from PVTech (OPV-TiO_2_-T).

Device fabrication

Perovskite solar cells (PSCs) were fabricated on FTO coated glass. FTO glass was ultrasonically cleaned with hellmanex, de-ionized water, acetone and isopropyl alcohol for 10 min. and dried with nitrogen. Before coating, oxygen plasma treatment was carried out to remove organic impurities and to activate the surface. Compact TiO_2_ layer was coated by spray coating technique. TiO_2_ solution was prepared by dissolving 3 ml of titanium isopropoxide in absolute ethanol. Another solution with 2 ml of acetylacetone in 6 ml of absolute ethanol was prepared. Acetylacetone solution was added to stirring titanium isopropoxide dropwise. The mixture was kept overnight by stirring at room temperature. Before spray coating stock solution was 1:1 diluted with absolute ethanol. FTO glasses were heated to 450 °C and coated by spray then kept for 3 min at the same temperature and cooled down to room temperature slowly.

Sepiolite layer

Sepiolite solution was prepared by dissolving in deionized water. The concentration of sepiolite was 1 mg/ml. The solution was kept in ultrasonic bath for 15 min at room temperature. Sepiolite layer was also coated onto TiO_2_ layer by spray coating at 130 °C. After coating the films were kept at 130 °C for 10 min. Final thickness of sepiolite layer was calculated to be around 300 nm.

Mesoporous TiO_2_

Mesoporous TiO_2_ layer was prepared by deposition of commercially provided TiO_2_ paste on compact TiO_2_ layer. TiO_2_ paste were statically spin coated at 6000 rpm for 60 s and annealed at 450 °C for 30 min by heating and cooling rate at 5 °C/min. Final thickness of mesoporous TiO_2_ layer was calculated to be 300 nm.

Perovskite layer

In this study, 3 different perovskite solutions were used. Well known GBL, ACN and DMF: DMSO solvent systems were used for perovskite solution.

Perovskite in GBL were prepared according to the previously reported procedure by Jeon et al. ^1^. PbI_2_ and MAI in gamma butyrolactone at a molar ratio of 1.23 M (PbI_2_ and methylammonium halide) was dissolved and kept for 2 hours at 80 °C. Then it was kept for 1 night at 60 °C. Perovskite layer was statically spin-coated at 1000 rpm for 10 s and then at 5000rpm 20s. Toluen was used as antisolvent and dropped when the spin rate reached to 5000 rpm. Obtained perovskite films were annealed at 100 °C for 30 min.

Perovskite in ACN solvent was prepared according to previously published procedure by Noel et al. ^2^. The precursor was prepared with the molar ratio of 1MAI: 1.06 PbI_2_ in ACN. ACN solution was exposed methylamine vapor by using argon flow that forms methyl amine bubbles in a glass bottle placed in ice bath until the obtaining clear and transparent perovskite solution. The final concentration of perovskite precursor was 0.5 M in acetonitrile. Perovskite layer was dynamically deposited by using ACN solution at 3000 rpm for 40 sec. Finally, the films were dried at 100 °C for 5 min.

Perovskite in DMSO:DMF solvent was prepared according to previously published procedure Zhang et al.^(^*^29^*^)^. The perovskite precursors, consisting of 922 mg PbI_2_ and 349.8 mg MAI were dissolved in 900 μL DMF and 100 μL DMSO. Then perovskite layer was deposited statically by spin-coating at 6000 rpm for 30 s. 200 μL of sec butyl alcohol as antisolvent was dropped at 7. second during spinning. At the second step, sec-butyl alcohol was added statically on perovskite coated layer and kept for 12 s and spin coated at 6000 rpm 30 s. Final perovskite films were dried at 100 °C for 30 min.

Hole transport layer

Spiro-OMeTAD solution consisting of 65 mg of spiro-OMeTAD, 20 μL of 4-tert-butyl pyridine, 70 μL of Li-TFSI (170mg mL-1 in acetonitrile) and 1mL of chlorobenzene was prepared and spin-coated at 4000 rpm for 30 s onto perovskite layer.

Finally, 100 nm of Au was thermally evaporated onto HTM layer by using a shadow mask. Active area of each electrode was calculated to be 0.023 cm^2^.

IV Characterizations

IV characterizations were carried out in nitrogen filled glove box system under AM 1.5 solar simulator by Keithley 2400 power source. Light intensities were measured with a calibrated KIPP&ZONEN pyranometer and calculated to be 80 mW/cm^2^.

Fig. S1. The photo of sepiolite in different solvents (upper), methanol, GBL, Pyridine, DMSO, Acetonitrile and DMF, and in water (lower).





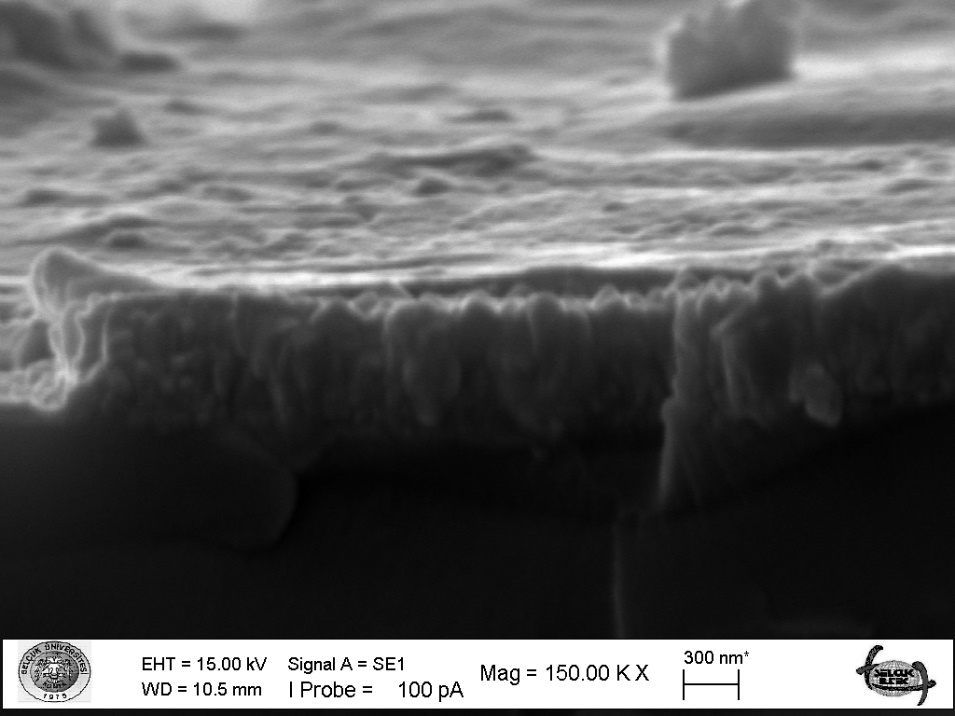


Fig. S2. Cross section SEM images of sepiolite layer. Sepiolite layer is observed to be around 400 nm.


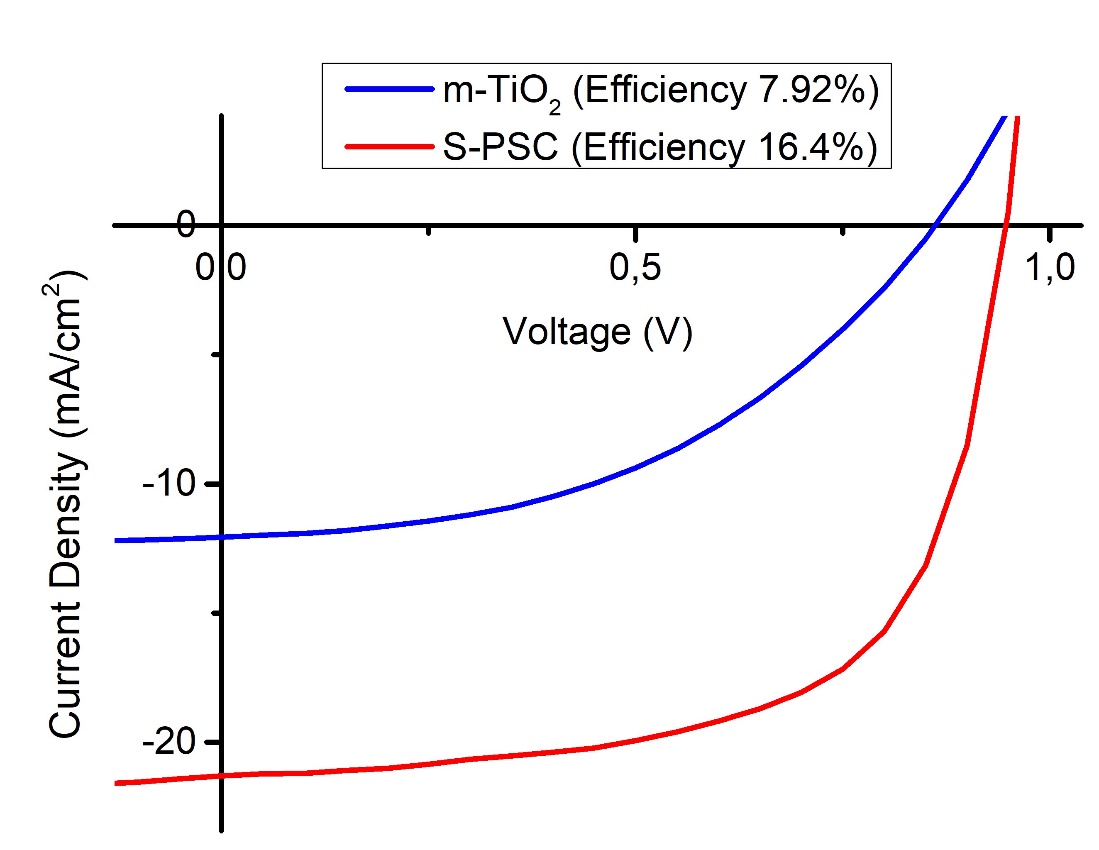


Fig. S3. IV curve of m-TiO_2_ and Sepiolite included PSC (S-PSC).


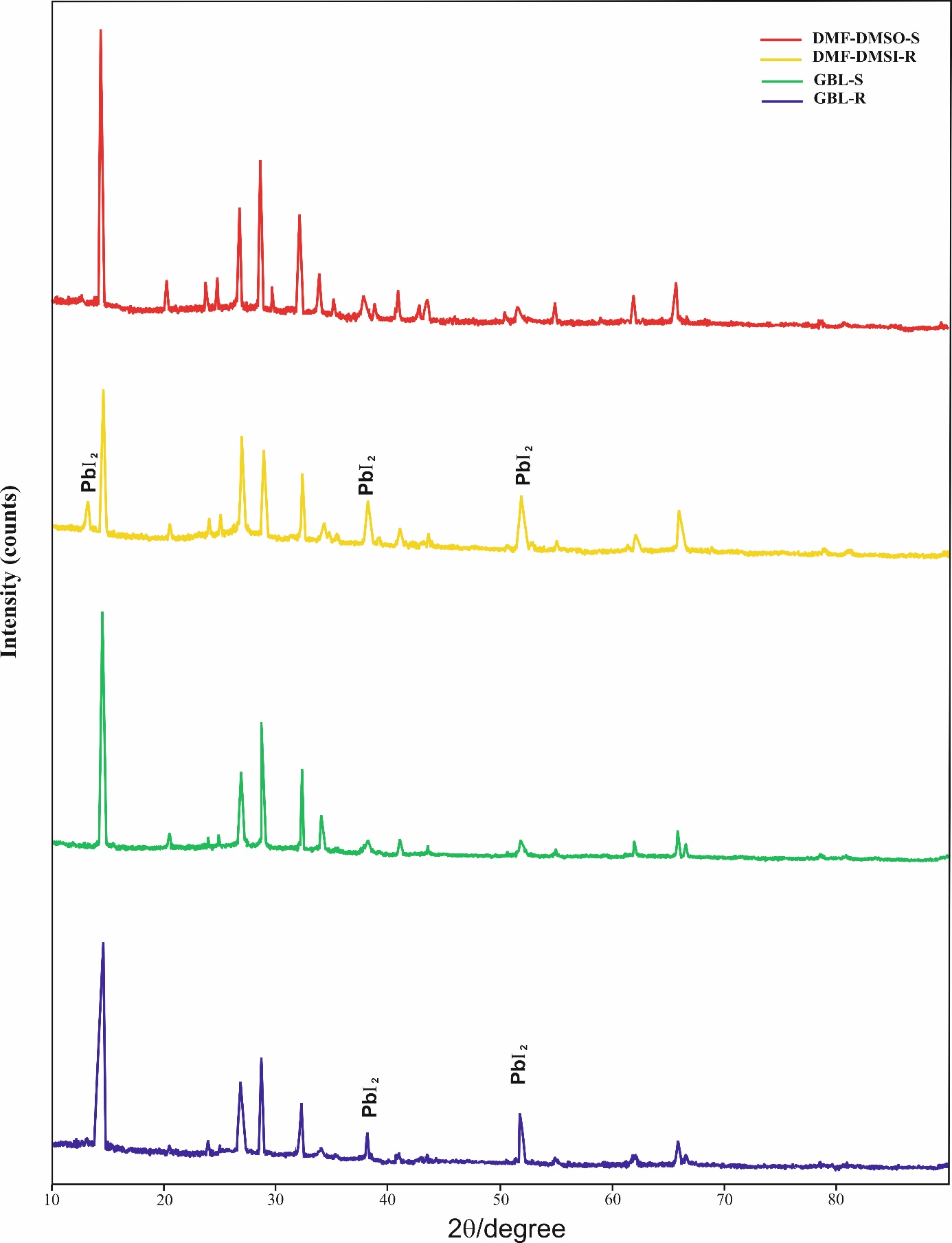


Fig. S4. XRD patterns of perovskite layer on Sepiolite (green line and red line ) and their reference (yellow and blue lines respectively). GBL and DMF:DMSO refer to solvent used for perovskite precursors.


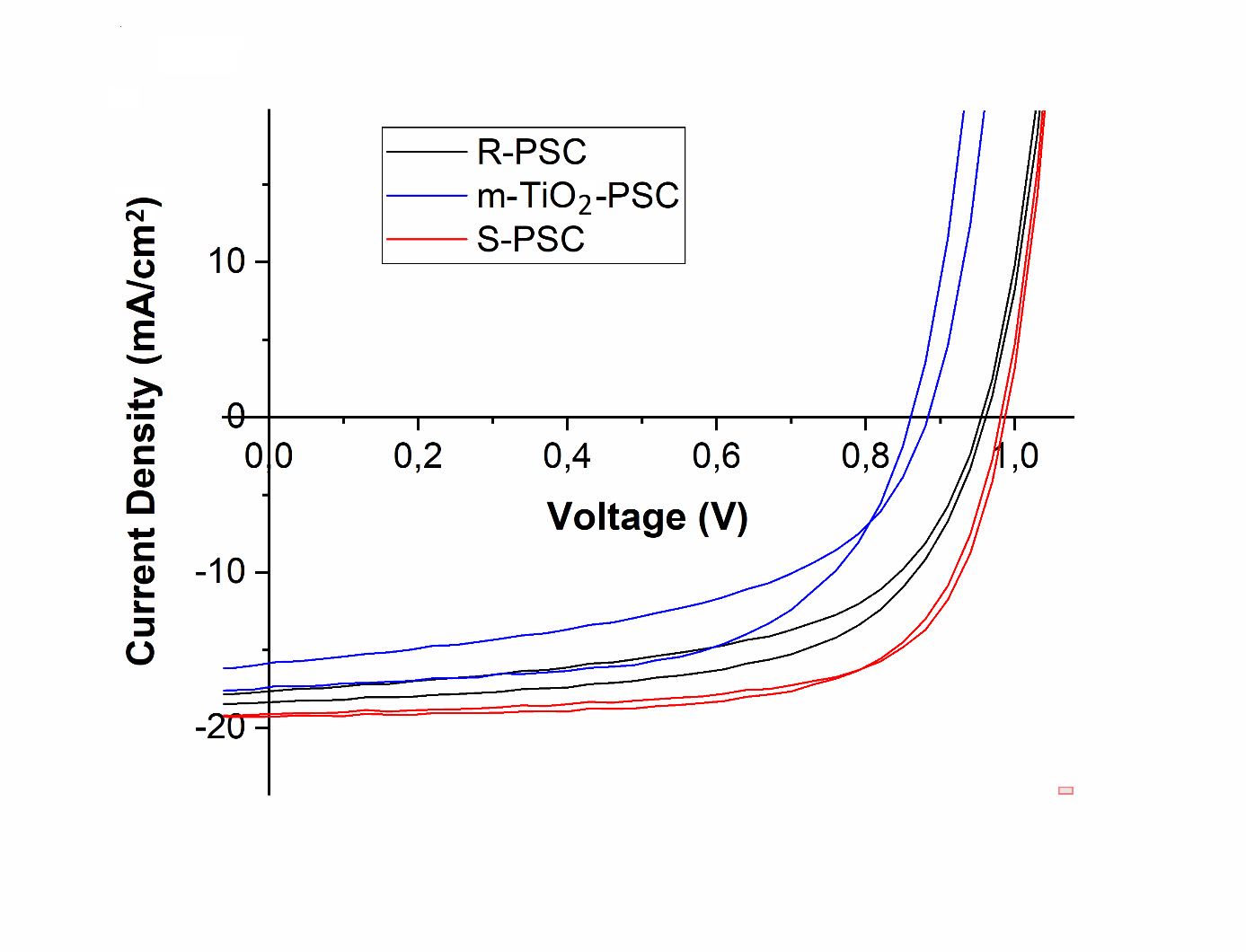


Fig. S5. Forward and revers scan of m-TiO_2_ (Blue line), Planar PSCs (Black)and Sepiolite included PSC (S-PSC).


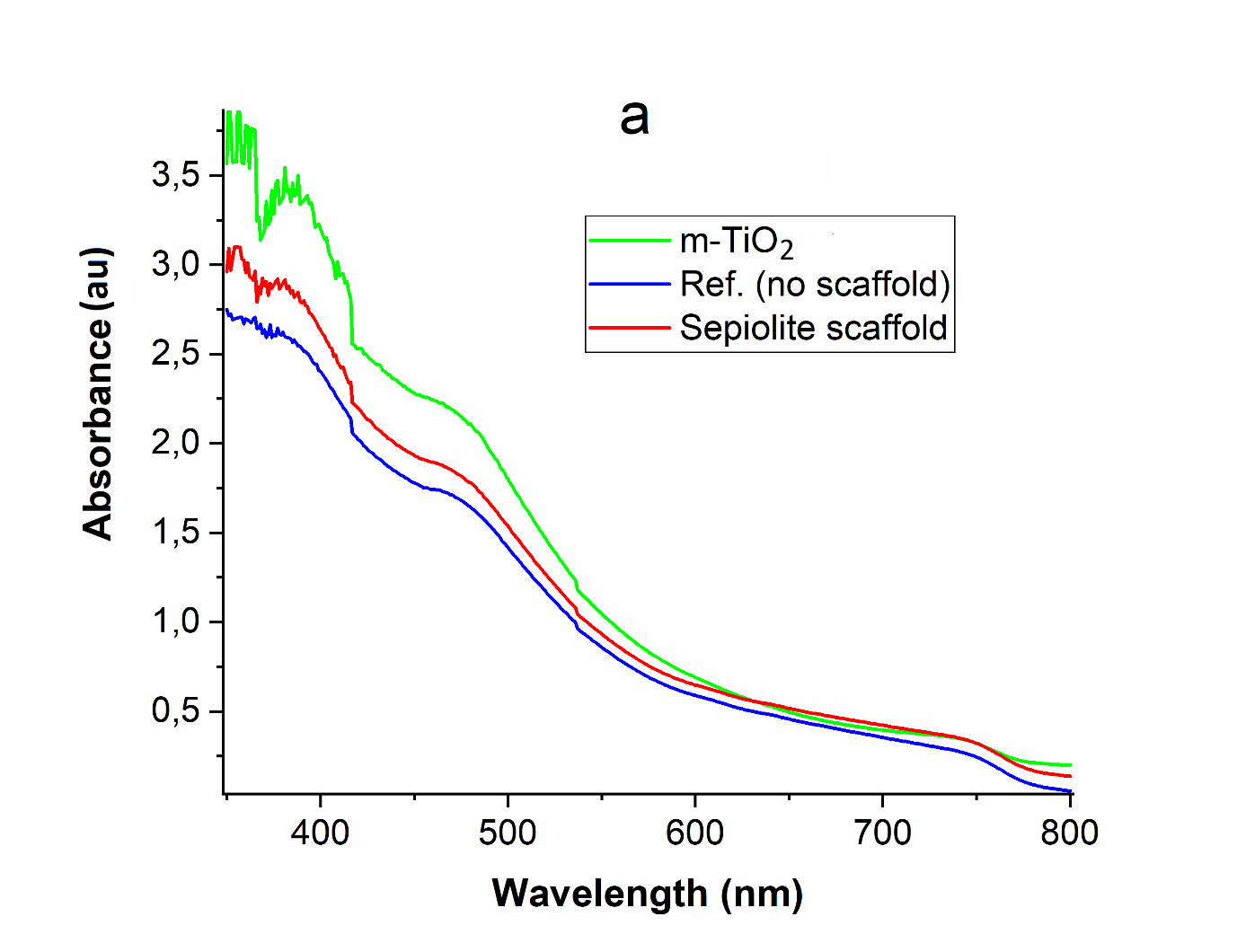


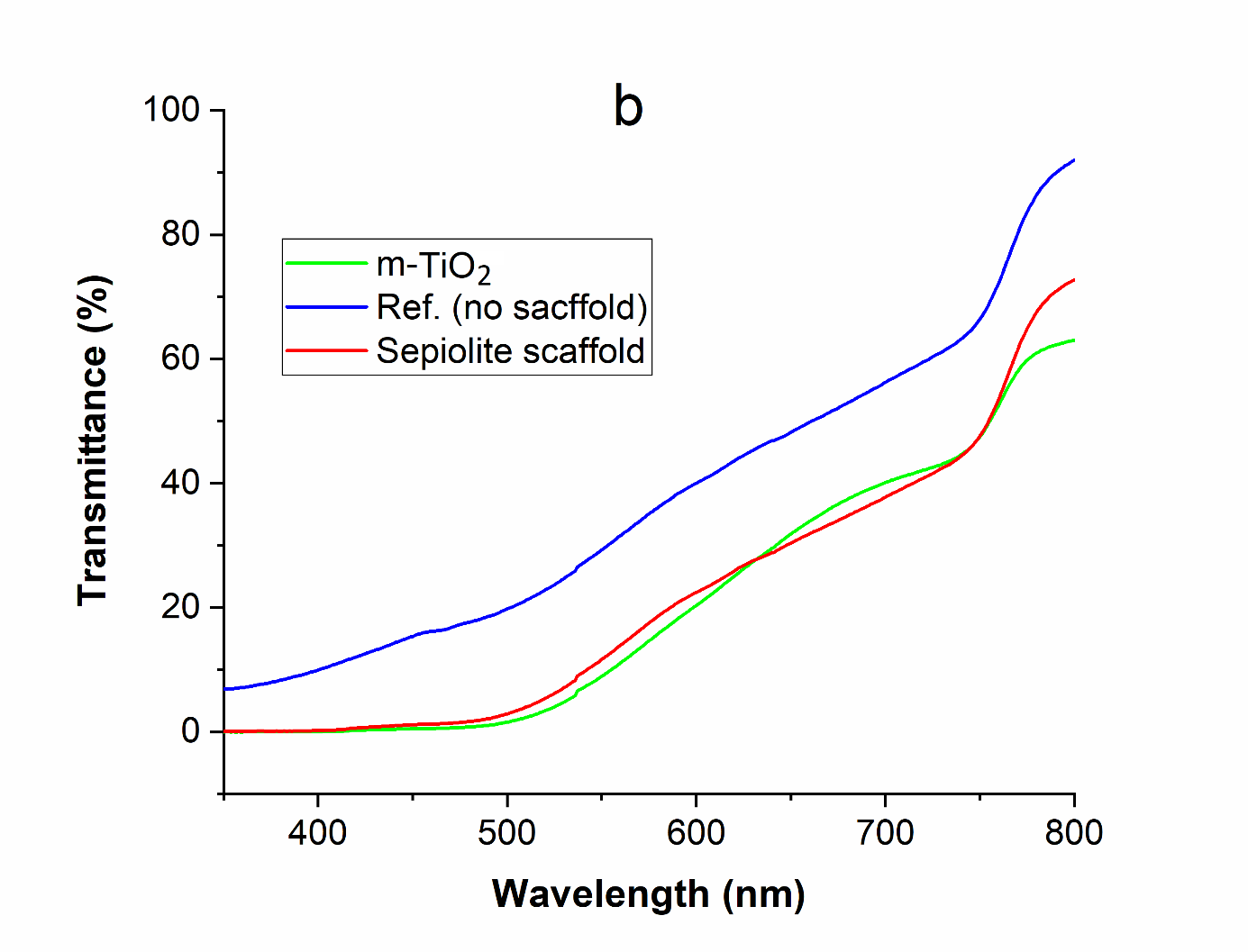


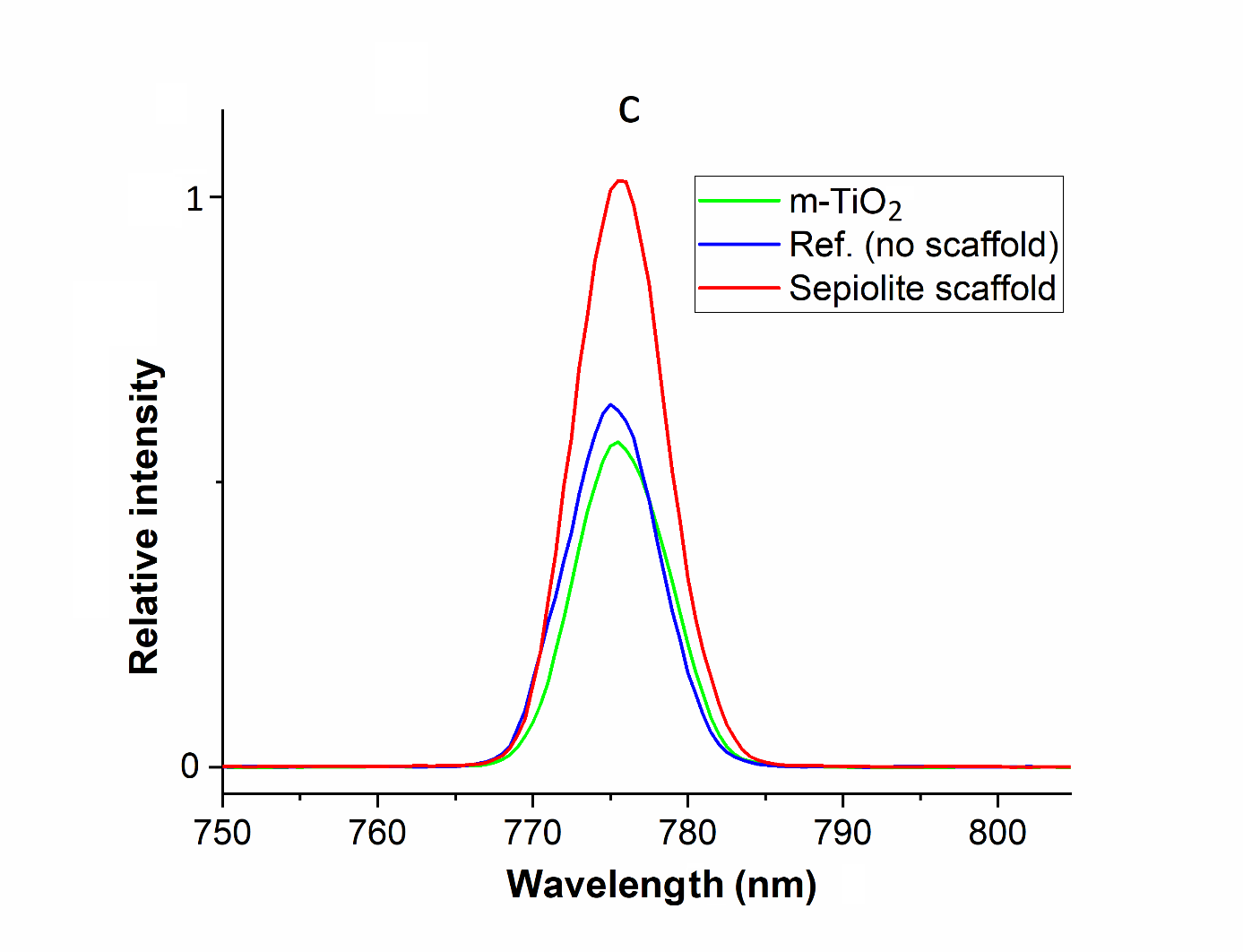





Fig. S6. Absorption (a), transmittance (b) and photoluminescence (c) of perovskite layer on mesoporous TiO_2_ (green line), sepiolite (red line) and without scaffold as reference (blue line) and (d) absorption and transmittance of sepiolite films.


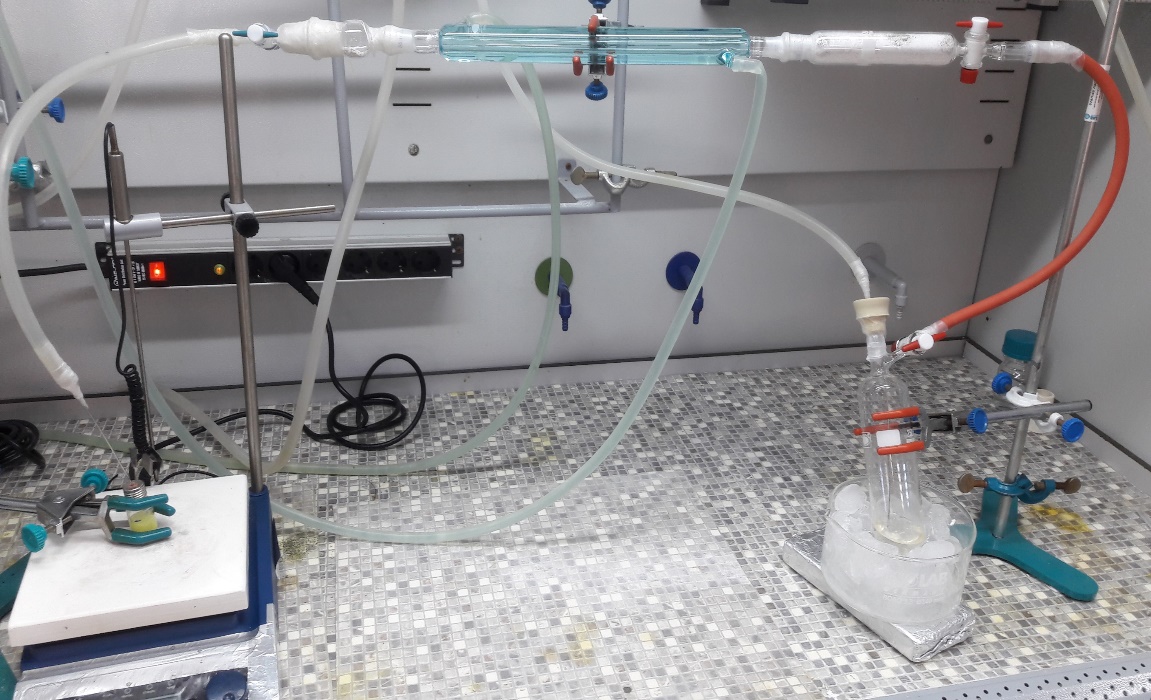


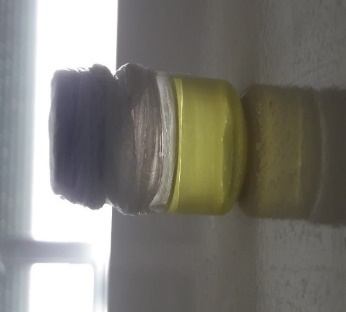


Fig. S7. The photo of preparation of perovskite solution in ACN under methyl amine gas flow (upper) and the photo of final solution (Lower).


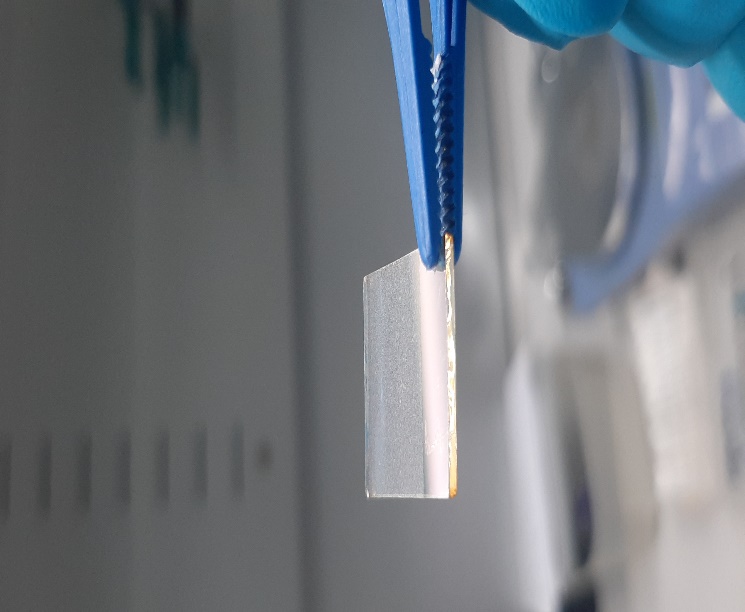

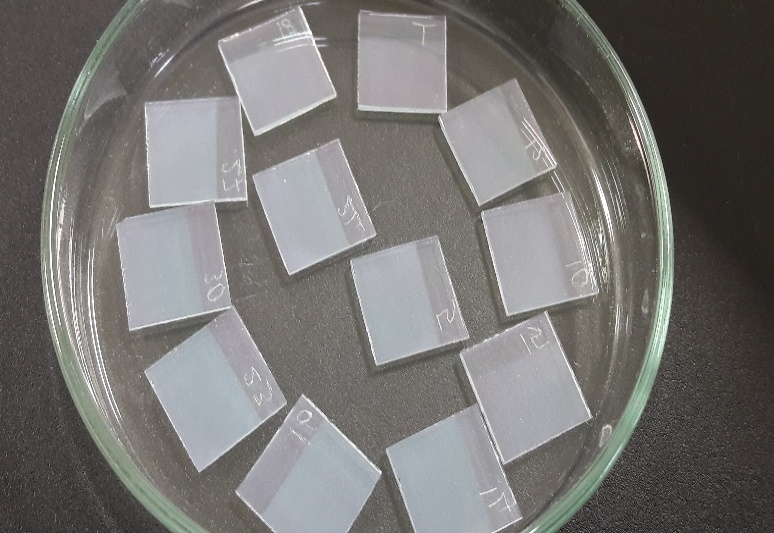


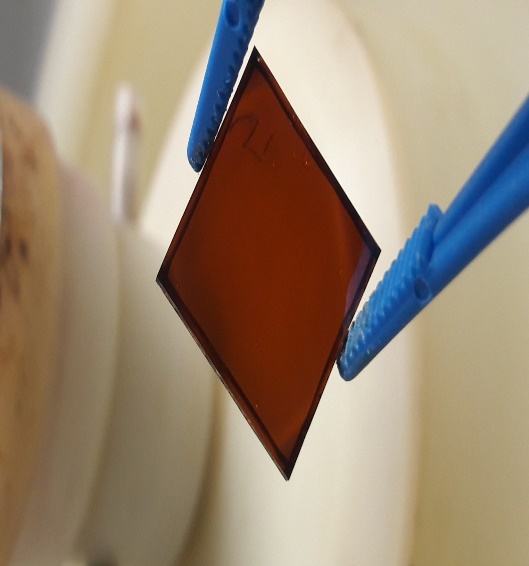

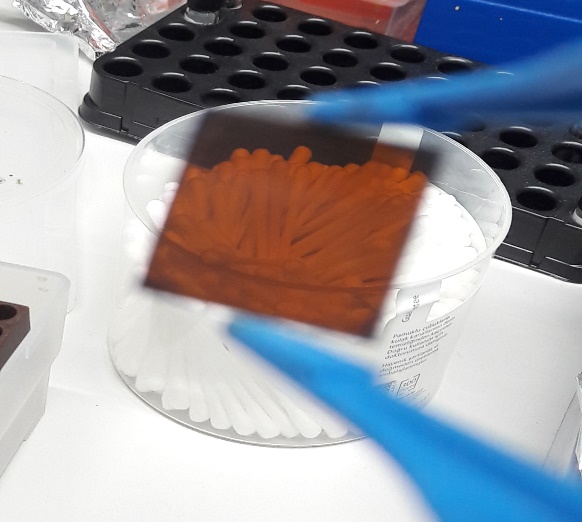

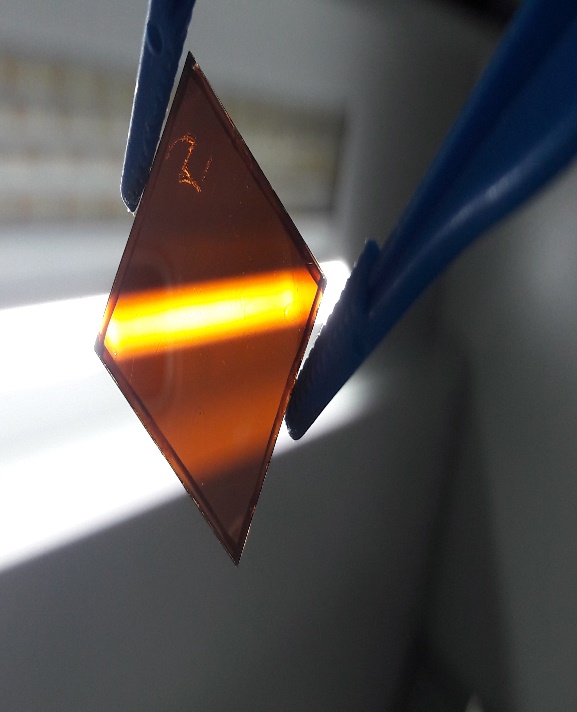


Fig. S8. Photos of sepiolite films and perovskite layers coated onto sepiolite films.


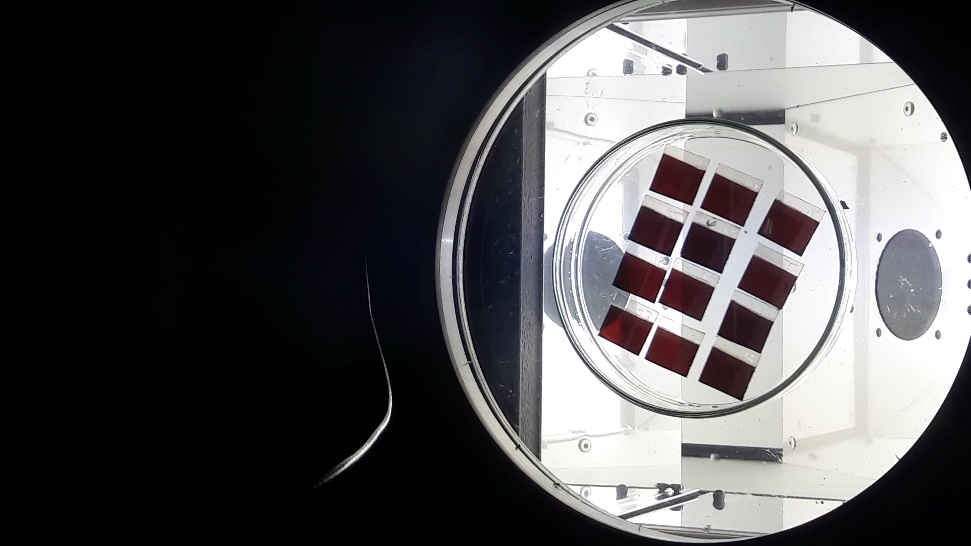

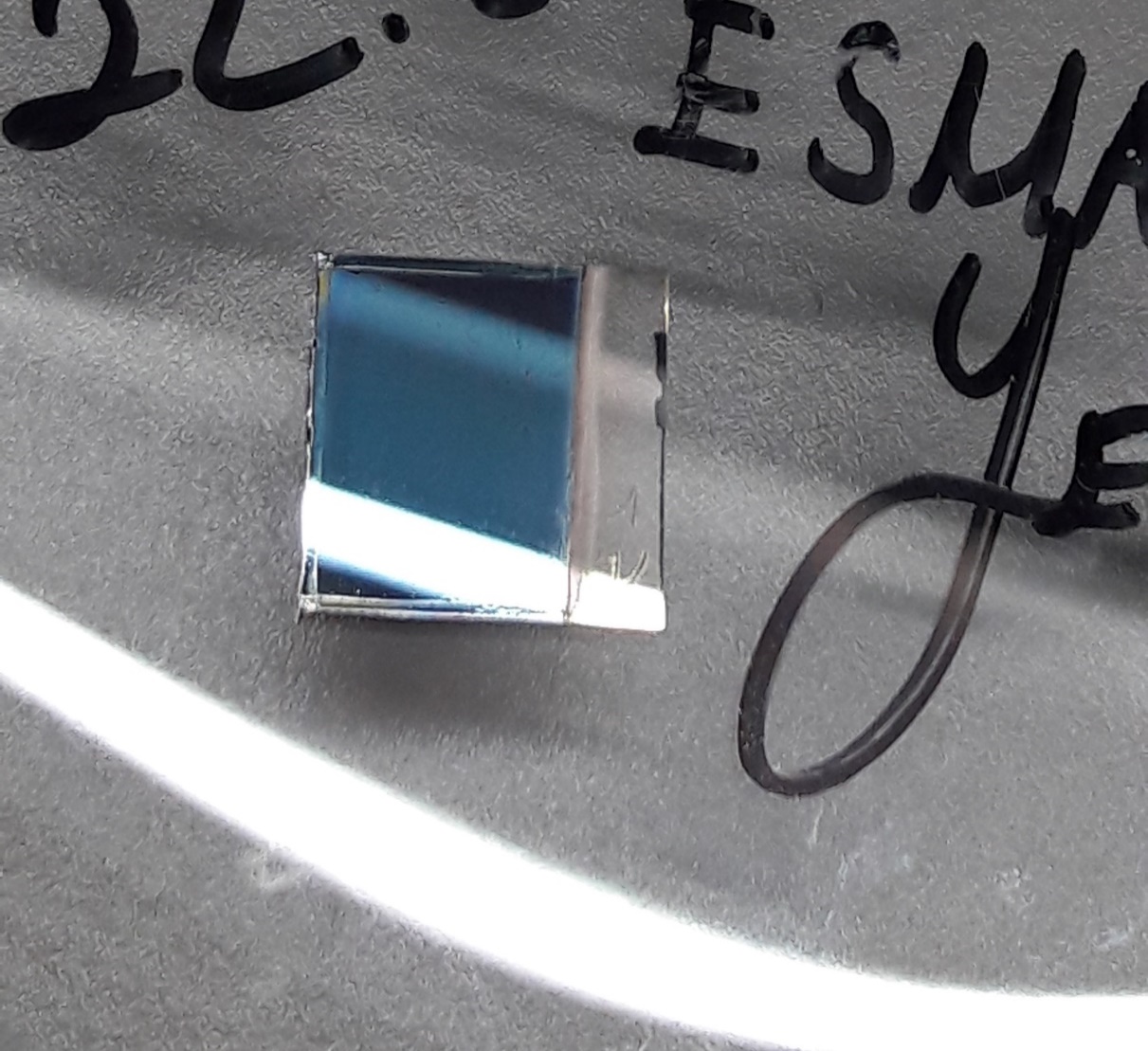


Fig. S9. Photos of HTM coated devices.


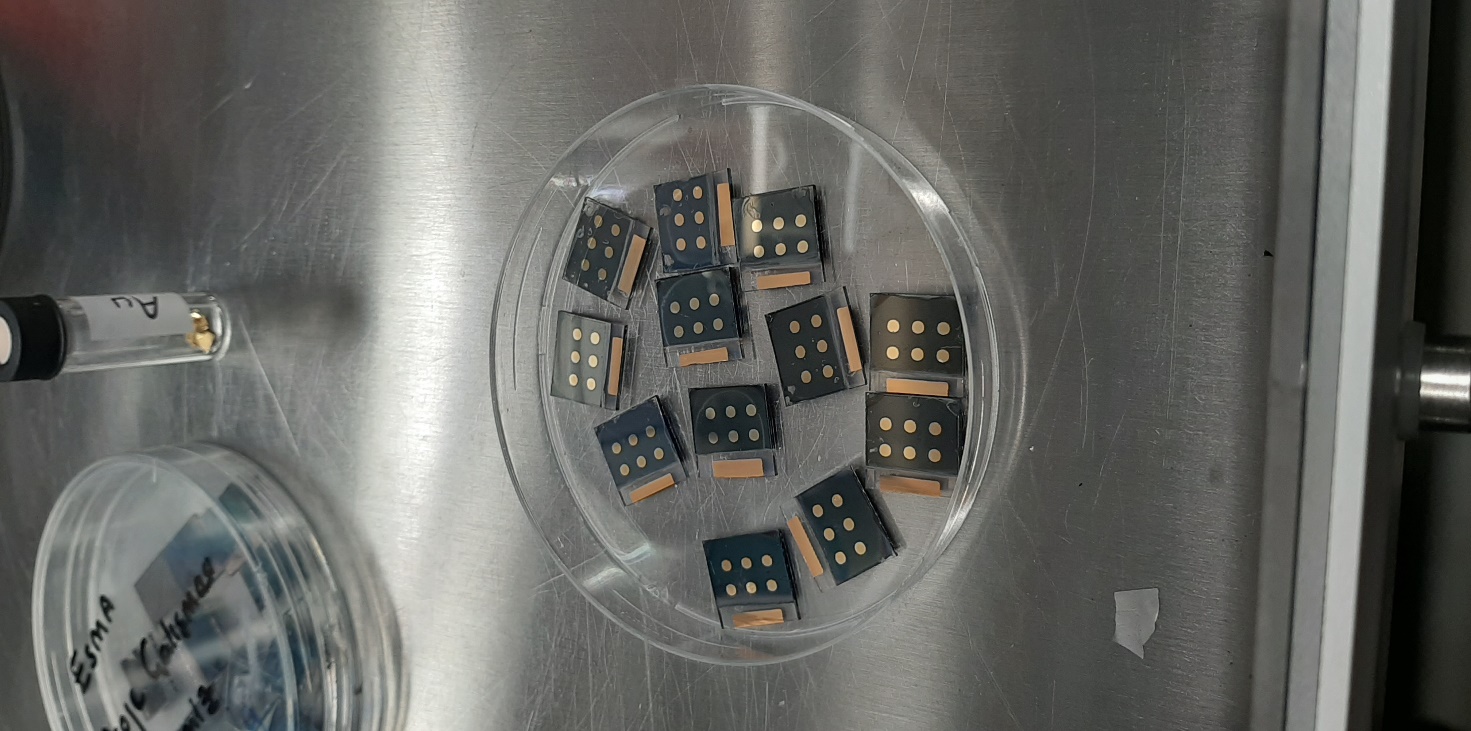


Fig. S10. Photos of final devices

**
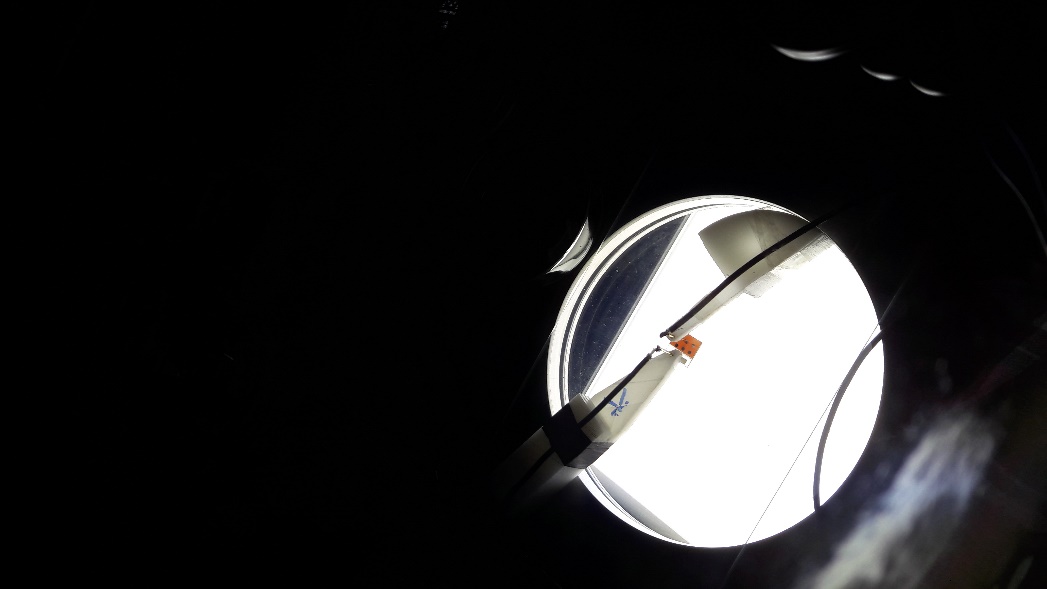
**

Fig. S11. Photo of measurement of PSCs under AM 1.5 solar simulator
